# Patterns of Remating Behaviour in *Ceratitis* (Diptera: Tephritidae) Species of Varying Lifespan

**DOI:** 10.3389/fphys.2022.824768

**Published:** 2022-02-07

**Authors:** Tania Pogue, Kevin Malod, Christopher W. Weldon

**Affiliations:** Department of Zoology and Entomology, University of Pretoria, Pretoria, South Africa

**Keywords:** lifespan, reproduction, sperm storage, trade-off, Tephritidae

## Abstract

Trade-offs between life-history traits offset the energetic costs of maintaining fitness in complex environments. *Ceratitis* species have been recorded to have long lifespans, which may have evolved in response to seasonal resource fluctuation. It is thus likely that reproductive patterns have evolved concomitantly as part of the trade-off between lifespan and reproduction. In this study, we investigated how reproductive patterns differ between *Ceratitis cosyra* (Walker) and *Ceratitis capitata* (Wiedemann; Diptera: Tephritidae), two species with different average and maximum lifespans. Females of both species were mated and patterns of female survival, fecundity, remating and sperm storage were tested. *Ceratitis cosyra* had a higher rate of survival and a lower fecundity when compared with the shorter-lived *C. capitata*, suggesting that both species exhibit a trade-off between lifespan and reproduction. Both species showed a similar and consistent willingness to remate, despite declines in sperm storage, suggesting that sperm alone does not fully inhibit remating. As expected, *C. cosyra* transferred high numbers of sperm during the first mating. However, sperm stores declined unexpectedly by 14 days. This indicates that males might transfer large ejaculates as a nuptial gift, that females then later degrade as a source of nutrients. Large declines in sperm storage may also indicate that females discard excess sperm stores due to the toxicity involved with storing sperm. These results do not suggest that patterns of sperm storage and remating align with lifespan and resource seasonality in these species, but a wider range of species needs to be assessed to better understand variation in *Ceratitis* mating systems.

## Introduction

The traits and characteristics that make up an organism’s lifestyle are known as its life-history (Braendle et al., 2011). Life histories evolve as a set of compromises between traits to best reflect evolutionary fitness in an organism’s natural environment (Wilbur et al., 1974; Partridge and Sibly, 1991). This evolutionary fitness is expressed through variation in individual survival and reproductive success (Dobson and Oli, 2007). Life-history traits are ultimately shaped by genetic drift and natural selection to maintain and maximise fitness in a given environment (Diamantidis et al., 2008).

Life-history trait evolution can be complex due to the variety of selective pressures that can be encountered (Schluter and Mcphail, 1992). Selective pressures rarely occur independently, with individuals being far more likely to face several interlinking selective pressures at once (Luhring et al., 2019). This can have complex influences on life-history traits (Bandara et al., 2019). Aspects of environmental variation, such as resource availability, act as a major selective pressure in the evolution of life-history traits (Suryan et al., 2009; Arlettaz et al., 2017; Craig et al., 2017). This can influence the survival and reproduction of species, which can in turn have significant impacts on population dynamics (Karell et al., 2009). For instance, some long-lived species may have evolved an increased lifespan as a mechanism to overcome environmental selective pressures, possibly to facilitate better synchronisation with host phenology (Malod et al., 2020).

This may be particularly true for frugivorous species due to the seasonality of many fruits. This has been observed in Ugandan fruit-feeding butterflies, where the nutritional ecology of adults, in terms of lack of year-round reliable fruit supply, may have contributed to the evolution of increased lifespan (Molleman et al., 2007). Extended lifespan observed in *Bactrocera opiliae*, a monophagous and univoltine species, has potentially evolved to survive to the next fruiting season without undergoing diapause (Fitt, 1981). Maintenance of bodily processes, both somatic and reproductive, is energetically costly and in the face of limited resources, individuals can often not afford to optimise both simultaneously (Kirkwood, 1977). As such, trade-offs between life-history traits maintain an evolutionary compromise in response to these selective pressures.

Life-history theory is centred on the concept that there is a demographic cost to reproduction (Williams, 1966). This cost is expressed as a priority in current reproductive value at the expense of future reproduction and survival (Messina and Slade, 2002; Harshman and Zera, 2007). Harshman and Zera (2007) proposed that metabolic and somatic regulation may be the cause behind reproductive costs, acting through direct processes of weakened immune function and decreased protection to toxic metabolites. Lifetime reproductive success in female insects is determined as a combination of egg production, fertility and lifespan, with longer lifespans being shown to result in higher lifetime reproductive success in females (Arnqvist and Nilsson, 2000). However, significant trade-offs have been repeatedly observed between reproduction and survival (Piper et al., 2005; Rogina et al., 2007; Carey et al., 2008).

Extended lifespans have been observed in several tephritid species (Duyck et al., 2012; Malod et al., 2020), with subsequent impacts on reproductive success (Carey et al., 1986, 2002, 2008; Müller et al., 2001; Carey and Molleman, 2010). Changes to reproductive success can impact a variety of factors, such as fertility, fecundity, sperm transfer and sperm storage patterns. In some tephritids, sperm numbers, and thus a female’s ability to produce viable offspring, undergo a general decline after copulation (Twig and Yuval, 2005). Female remating is thus required to replenish the inadequate provision of sperm provided by males during the first mating event (Mossinson and Yuval, 2003; Gavriel et al., 2009). Female remating in tephritids has been found to be regulated by a number of factors that differ among tephritid species, such as mating duration, male condition and transfer of the ejaculate (Kuba and Itô, 1993; Vera et al., 2002, 2003; Gavriel et al., 2009). As a result, patterns of reproduction and lifespan may differ between species within the same genus. For instance, when held under similar conditions, *C. cosyra* has a longer average lifespan (160 days) than *C. capitata* (110 days; Malod et al., 2020). In conjunction with this, *C. cosyra* also transfers higher numbers of sperm to females during mating and has substantially longer copula durations when compared to *C. capitata* (Churchill-Stanland et al., 1986; Field and Yuval, 1999; Bertin et al., 2010; Roets et al., 2018). Within southern Africa, the longer-lived *C. cosyra* has a more restricted host range when compared to *C. capitata* (De Villiers et al., 2013). It is thus likely that these two species differ in other aspects of their reproductive biology as dictated by the lifespan-reproduction trade-off and availability of oviposition resources.

In this study we aimed to explore the lifespan-reproduction trade-off and its effects on two *Ceratitis* species of varying lifespan. More specifically, we aimed to compare fecundity, fertility and patterns of sperm transfer between the two species. To do this, we compared and analysed patterns of mating, fertility, sperm storage and survival between *C. cosyra* and *C. capitata*. *Ceratitis cosyra* transfers large numbers of sperm during mating (Roets et al., 2018) and show a decrease in survival when selected for early age of female reproduction (Malod et al., 2020). As a result of this, we predicted that males of the longer-lived *C. cosyra* would transfer more sperm during first mating as a way for females to maintain reproductive potential throughout their life. Additionally, we predicted that *C. cosyra* would have lower fecundity and fertility compared to *C. capitata*, as part of the trade-off between lifespan and reproduction.

## Materials and Methods

### Study Species and Fly Husbandry

Two *Ceratitis* species (Diptera: Tephritidae), that have differing average lifespans, were used. When held under similar conditions, *C. cosyra*, has an average lifespan of 160 days, whereas *C. capitata* has a shorter lifespan of 110 days on average (Malod et al., 2020). Flies were obtained from pre-established cultures at the University of Pretoria, South Africa. The *C. cosyra* culture was originally obtained from Citrus Research International, with wild flies being added yearly to introduce new genetic material and was maintained in the lab for seven generations before flies were collected for this study. The *C. capitata* culture was originally sourced from coffee plants grown on the ARC Bugershall Research Station near Hazyview, Mpumalanga, South Africa, and was maintained in the lab for nine generations before flies were collected for this study. Although this number of generations of colonisation may be enough for laboratory adaptation effects to occur, the generation number is similar between species and so laboratory adaptation would likely have the same effect for both species. The flies were kept in a climate-controlled room at an average temperature of 22.0 ± 1.6°C for *C. capitata* and 22.2 ± 3.0°C for *C. cosyra* with 40–60% relative humidity and a 14:10 h LD photoperiod. A 1-h dawn and dusk period was simulated for the first and last hour of the light cycle, using fluorescent tubes, to provide adequate mating conditions.

Eggs from both species were collected from culture flies older than 10 days by making an oviposition substrate available to the culture. The oviposition substrate comprised a 125-ml plastic container, with a 63 mm diameter opening, containing a folded paper towel soaked in water and 3 ml of guava concentrate (Fruitree Superfruit Guava, Pioneer Foods, Tyger Valley, South Africa). This was then covered with a double layer of laboratory film (Parafilm ‘M’; Bemis Company Inc., Oshkosh, Wisconsin) and was pierced with an entomological pin approximately 50 times. Eggs laid on the oviposition substrate were collected from the container by rinsing it with distilled water into a glass dish, which were then collected with a Pasteur pipette and transferred to another 125-ml container filled with standard carrot-based larval diet (Citrus Research International, Nelspruit, South Africa). To control for the impact of larval competition, approximately three eggs per 1 ml of larval diet were inoculated. Inoculated diets were placed in well-ventilated plastic containers with sand lining the bottom and were placed in a climate room (same conditions as above). Pupae were sifted from the sand after 10 days and transferred into a Petri dish after which they were placed into a modified 5-L cage. The cage comprised a clear plastic container, with voile fabric replacing the lid, and one side cut open and replaced with insect screen for appropriate ventilation.

Adults were sexed and separated within 48 h of emergence, based on the presence or absence of an ovipositor. Females used in the study were placed individually in cages for the first mating procedure and kept in the cages until the end of data collection. The cage comprised two 125-ml containers, one stacked within the other, with the base of the upper container removed. Each cage was covered with insect screen and secured with rubber bands. Flies had unrestricted access to white sugar and hydrolysed yeast (Yeast Extract Powder; Biolab; Merck, Germany), placed in separate microcentrifuge caps as a food source. Distilled water was available from a 200 μl pipette tip that was loosely sealed with putty-like adhesive (Prestik; Bostik, South Africa). The experimental design outlined below was repeated for each species.

### First Mating Procedure

At the age of sexual maturity, 300 *C. capitata* and 329 *C. cosyra* females were placed individually in the 125-ml cages. *Ceratitis capitata* was mated at 9 days old (Arita, 1982) and *C. cosyra* was mated at 10 days old (Manrakhan and Lux, 2006). A single virgin male fly was introduced to each cage 90 min before artificial sunrise and sunset, for *C. capitata* and *C. cosyra*, respectively. This was required because *C. capitata* mate during the day with a peak between 10:30 and 13:00 h (Prokopy and Hendrichs, 1979) whereas *C. cosyra* mate at dusk (Manrakhan and Lux, 2009). A 1-h observation period was conducted during dawn for *C. capitata* and during dusk for *C. cosyra*, thereafter observations were made every 30-min until midday and late evening, respectively. The occurrence of mating was noted, along with the time that mating began and ended, and the time taken for the pairs to mate from the beginning of artificial dawn or dusk. For both species, first mating, as well as remating, fecundity, survival and sperm storage (described below) were assessed at 25.1 ± .9°C and 60 ± 7% relative humidity.

### Remating Study

On days 7, 14, 21, 28, 35 and 42 after first mating, a new virgin male at peak age of reproductive performance (9–10 days old) was introduced to 97 mated *C. capitata* and 93 mated *C. cosyra* females in their individual cages to provide an opportunity to remate. Remating procedures followed the same procedure as the first mating event. Mating latency, mating propensity and copula duration were recorded at each remating event, as described in the procedure for first mating. When a pair mated multiple times within the observation period, the beginning and end of each copulation were noted and the total mating duration was used. Males that were offered during the remating opportunities were not reused for further remating attempts and were removed from the study regardless of the result of the remating event. However, females that remated remained in the study and were given subsequent repeated opportunities to remate each week.

### Fertility, Fecundity, Survival and Sperm Storage

Fertility for 114 mated *C. capitata* and 140 mated *C. cosyra* females, that were not given the opportunity to remate, was determined by counting the number of eggs laid per female. The number of eggs laid per female was compared to the number of first instar larvae that emerged from the eggs, as a measure of fertility. These counts were conducted at days 2, 8, 15, 22, 29, 36 and 43 after the first mating, with the oviposition substrate available for the full week. The oviposition substrate for each female was then removed and a fresh oviposition substrate was added to each individual cage. Eggs were counted within 2 days of collection of the oviposition substrate. The bottom of each container was also inspected for eggs, which were added to the count. The oviposition substrate comprised a black screw top lid (volume 5 ml, diameter 32 mm), containing approximately 3-ml of a 1:50 guava concentrate (Fruitree Superfruit Guava, Pioneer Foods, Tyger Valley, South Africa) to distilled water ratio. The oviposition substrate was covered in a double layer of Parafilm (Parafilm ‘M’; Bemis Company Inc., Oshkosh, Wisconsin), pierced with a pin approximately 15 times and placed in the bottom of each cage.

Mortality of these flies was recorded daily.

A total of 50 mated females for both species was randomly selected during the experiment for assessment of sperm storage and were also provided with oviposition substrates as described above. On the day after first mating and at day 7, 14, 28 and 42 after the first mating, sperm storage counts were conducted from 10 of the total 50 females set aside for sperm storage assessment. Each count was from 10 randomly selected females that were dissected under a stereo microscope to remove the spermathecae. Each spermatheca was placed separately on a microscope slide in 15-μl drops of distilled water. The spermathecae were crushed with an entomological pin and stirred vigorously for 30 s to release stored sperm. Coverslips (22 × 22 mm) were placed over the crushed spermathecae and sealed in place with clear nail varnish after the slide had dried. Sperm was counted in 16 evenly spaced fields of view, under a phase-contrast microscope (BX43; Olympus Corporation), using 100 x magnification. This comprised 12.56% of the slide, which was then multiplied by 7.957 to obtain the total number of sperm per spermatheca (Taylor et al., 2000). Patterns of sperm storage asymmetry were determined by comparing the difference in number of sperm from each spermatheca.

### Statistical Analysis

All statistical analyses were performed in the R v. 4.0.3 statistical environment, running in RStudio v. 4.0.3 (R Core Team, 2020). Survival analysis was performed using a log rank test to determine patterns of mortality in mated females in relation to species and time since mating. The survival analysis model was built using the ‘survfit’ function from the ‘survival’ package (Therneau, 2020). Generalised linear mixed models, with fly identity as a random effect (due to repeated measurements), were used to analyse the effects of species and time since mating on fecundity, with a negative binomial distribution, and on fertility, with a binomial distribution. Due to the null variance of the random effect, generalised linear models were used to analyse mating propensity and mating latency, with binomial and gaussian distributions, respectively.

Data for mating duration, sperm storage asymmetry and total sperm storage were found to be overdispersed. We determined minimum adequate models for these analyses based on the lowest value for quasi Akaike’s information criterion in order to account for overdispersion within the models. Generalised linear models, excluding fly identity as a random effect due to null variance, were used for all minimum adequate models, with quasi-Poisson, quasi-binomial and quasi-Gaussian distributions used for mating duration, sperm storage asymmetry and total sperm storage, respectively.

Models were built using the ‘glm’ or ‘glmer’ functions from the ‘lme4’ package (Bates et al., 2015). Tukey’s honest significant difference test was used to conduct intraspecific pairwise *post-hoc* comparisons where necessary. This was conducted using estimated marginal means from the ‘emmeans’ function and the package of the same name (Russel, 2020).

## Results

### Remating

**Figure 1:**
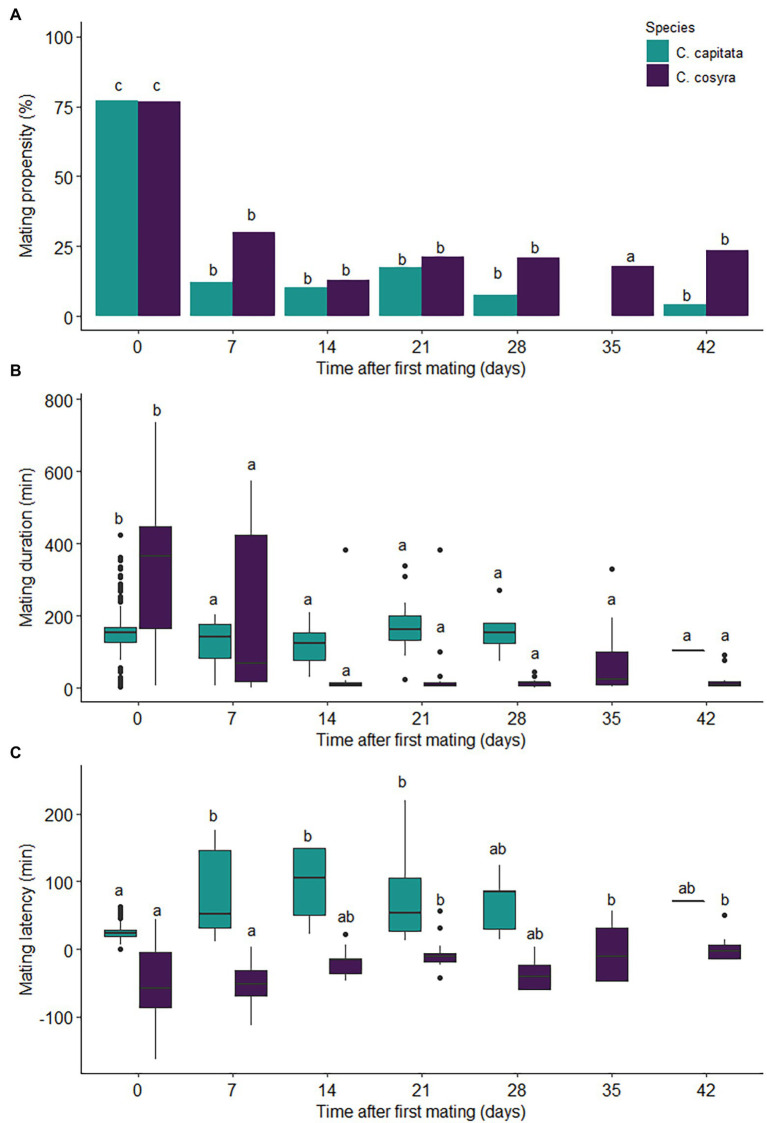
Mating behaviour of *C. capitata* (*n* = 97) and *C. cosyra* (*n* = 93) females kept individually in containers and given weekly remating opportunities with a virgin male of peak reproductive age. The values displayed are the **(A)** mating propensity, **(B)** mating duration and **(C)** mating latency of remated pairs. Time intervals within species with the same lowercase letter are not significantly different (Tukey’s HSD *post-hoc* tests, *α* = .05).

Time since mating (χ^2^ = 377.46, df = 6, *p* < .001) and the interaction between time and species (χ^2^ = 25.63, df = 6, *p* < .001) had a significant effect on mating propensity, with mating propensity showing a significant decline after the first mating for both species (Figure 1A). Mating propensity was significantly higher for the first mating for both *C. capitata* and *C. cosyra*. Thereafter mating propensity declined and remained consistent across subsequent mating opportunities for both species, with the percentage of females that remated remaining below 30.11% for all remating opportunities (apart from *C. capitata* at 35 days after first mating, when no remating occurred; Figure 1A). Throughout all mating opportunities, species had no significant effect on mating propensity (χ^2^ = .01, df = 1, *p* = .905). Of the females that remated, 16.28% of *C. capitata* and 25.49% of *C. cosyra* females remated more than once.

Time since first mating was the only term retained in the minimum adequate model and had a significant effect on mating duration (χ^2^ = 152.03, df = 6, *p* < .001). The duration of the first mating, an average of 150 ± 66 min for *C. capitata* and 331 ± 176 min for *C. cosyra*, exceeded that of all rematings for both species (estimate = 5.501, *p* < .001; Figure 1B).

Mating latency was significantly affected by an interaction between species and time since first mating (χ^2^ = 10.02, df = 1, *p* < .001; Figure 1C). Mating latency increased slightly after the first mating for *C. capitata* before decreasing back to similar proportions to that of the first mating (26 ± 13 min) at 28 days later (Figure 1C). In contrast, mating latency remained relatively unchanged after the first mating for *C. cosyra* (50 ± 54 min) until day 21, where a slight increase in mating latency was then observed (Figure 1C).

### Survival, Fecundity and Fertility

**Figure 2:**
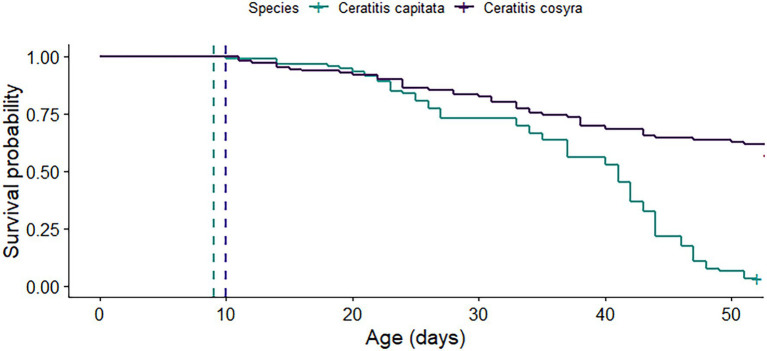
Survival curves of *C. capitata* and *C. cosyra* females that were mated at 9 (blue dashed line) and 10 days old (purple dashed line) respectively. The curves represent 114 mated *C. capitata* and 140 mated *C. cosyra* that were kept in individual containers and were provided with fresh oviposition substrates each week. Survival of both species was recorded until 50 days postemergence.

*C. cosyra* had a significantly higher lifespan than *C. capitata* (χ^2^ = 60.7, df = 1, *p* < .001). By 52 days after mating, only 3.23% of *C. capitata* had survived, in contrast with 61.81% for *C. cosyra* (Figure 2). These survival estimates exclude the cohort of females set aside for dissections that were used to determine sperm storage patterns.

**Figure 3:**
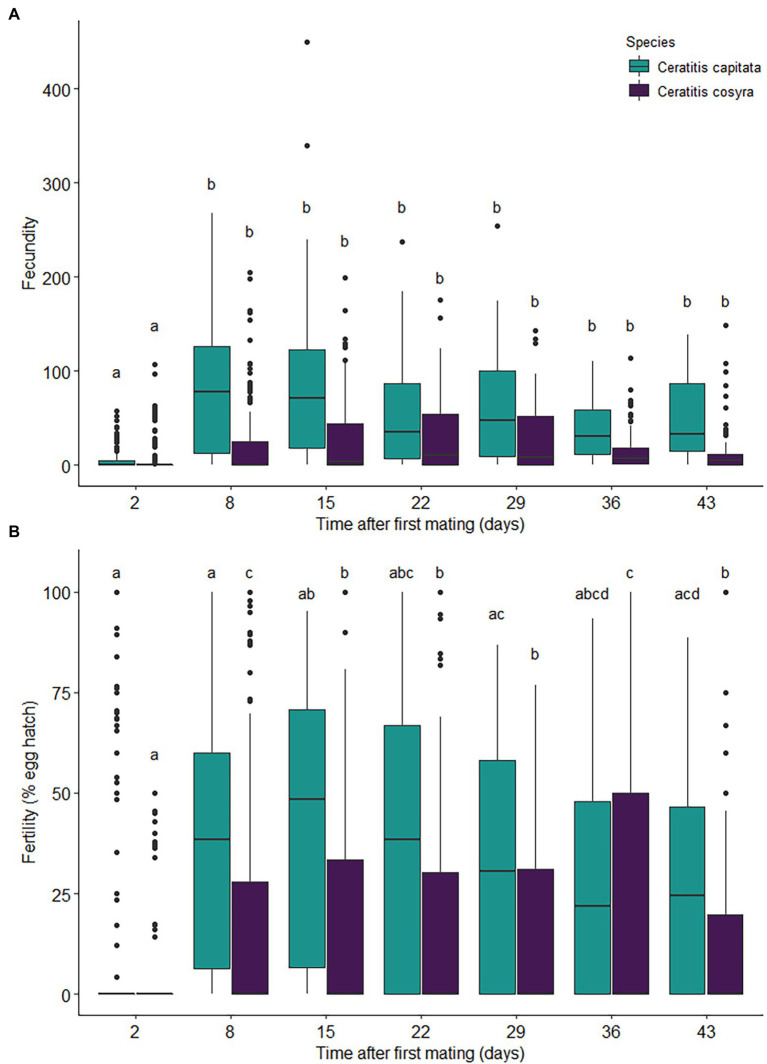
Reproductive effort of *C. capitata* (*n* = 114) and *C. cosyra* (*n* = 140) females kept individually in containers over a 43-day period after mating with a virgin male of peak reproductive age and provided with a fresh oviposition substrate each week. The values displayed are the **(A)** number of eggs laid (fecundity) and **(B)** percentage of eggs that hatched (fertility). Time intervals within species with the same lowercase letter are not significantly different (Tukey’s HSD *post-hoc* tests, *α* = .05).

There was a significant interaction between time since mating and species on fecundity (χ^2^ = 16.46, df = 6, *p* = .011), with the main effect of time since mating also having a significant effect on fecundity (χ^2^ = 184.86, df = 6, *p* < .001). Fecundity varied greatly in both species, but with *C. capitata* laying significantly more eggs overall (χ^2^ = 7.85, df = 1, *p* = .005; Figure 3A). For both species, fecundity was significantly lower 2 days after mating when compared with later days (Figure 3A). Thereafter, *C. capitata* laid significantly more eggs than *C. cosyra* at each time interval (Figure 3A).

Time since mating (χ^2^ = 111.90, df = 6, *p* < .001) and its interaction with species (χ^2^ = 144.25, df = 6, *p* < .001) significantly affected fertility. In *C. capitata*, fertility increased until 15 days after mating, where it remained consistent until 22 days after mating and then began to decline (Figure 3B). The fertility observed for *C. capitata* 29 days after mating were similar to those seen 8 days after mating and showed further declines thereafter (Figure 3B). Fertility in *C. cosyra* showed an increase until 8 days after mating and then decreased and remained consistent until 29 days after mating (Figure 3B). Fertility for *C. cosyra* increased again 36 days after mating before decreasing back to previous levels (Figure 3B). Fertility differed significantly between species (χ^2^ = 19.03, df = 1, *p* < .001), with a higher proportion of eggs hatching in *C. capitata* than in *C. cosyra* (Figure 3B).

### Sperm Storage

**Figure 4:**
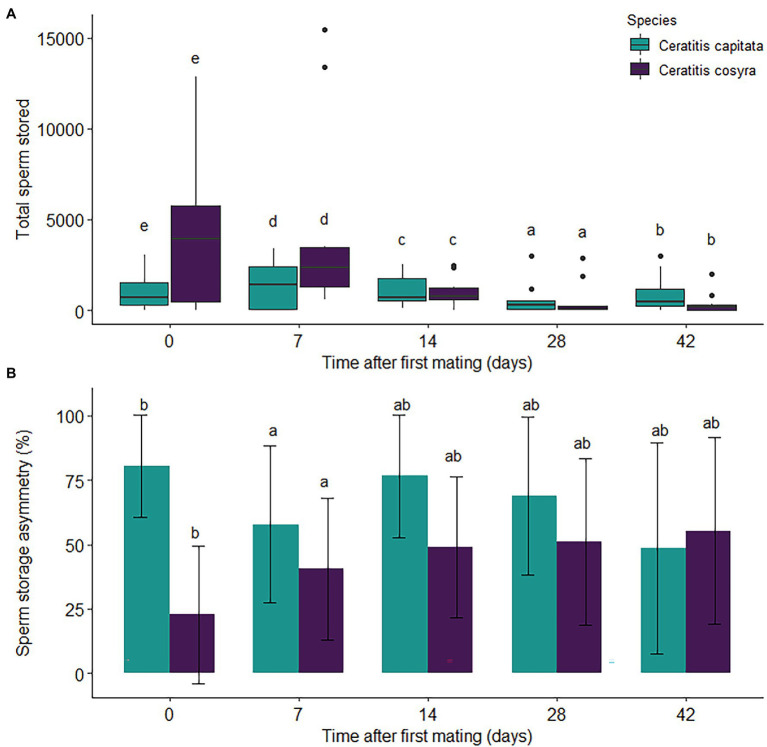
Sperm storage by mated female *C. capitata* and *C. cosyra* mated once and kept individually in containers. The values displayed are the **(A)** total sperm stored and **(B)** mean (±1 s.e.) percentage sperm storage asymmetry of females over a 42-day period after mating. For both species, each time interval represents 10 dissected females. Time intervals within species with the same lowercase letter are not significantly different (Tukey’s HSD *post-hoc* tests, *α* = .05).

Overall, higher numbers of sperm were transferred to *C. cosyra* than to *C. capitata* females (χ^2^ = 2041.2, df = 1, *p* < .005), with 4,117 ± 4,057 and 999 ± 995 spermatozoa being stored directly after mating, respectively. Time since mating had a significant effect on the total sperm stored by females (χ^2^ = 3357.2, df = 4, *p* < .005). Although general declines in sperm storage occurred in both species (Figure 4A), a large decline in sperm storage was seen in *C. cosyra* 14 days after mating (coefficient = 1.472, *p* < .001).

The minimal adequate model for sperm storage asymmetry included the main effects of time since mating and species. Sperm storage asymmetry was significantly lower in *C. cosyra* than in *C. capitata* (χ^2^ = 21.352, df = 1, *p* < .001), observed at 32.5 ± 26.6% and 75.4 ± 19.3%, respectively, directly after mating. For both species, time since mating had a significant effect on sperm storage asymmetry (χ^2^ = 13.748, df = 4, *p* = .008), with sperm storage asymmetry decreasing 7 days after first mating (coefficient = .509, *p* = .030; Figure 4B).

## Discussion

In this study we aimed to determine how reproductive traits and patterns vary in two *Ceratitis* species of different lifespan. A higher survival and lower rate of fecundity and fertility were observed in *C. cosyra* than in the more generalised *C. capitata*, with the peak in fecundity and fertility in *C. cosyra* occurring later than that of *C. capitata*. Despite differences in lifespan, *C. capitata* and *C. cosyra* exhibited similar mating propensities, with both mating propensity and mating duration declining after first mating. Overall sperm storage declined after the first mating, with fertility and fecundity initially increasing over time after first mating as a result. *Ceratitis cosyra* initially stored more sperm than *C. capitata*, but there was a large decline in sperm storage occurring 14 days after mating. Additionally, sperm was initially found to be stored more evenly across spermathecae in *C. cosyra* than in *C. capitata*.

Many life-history traits form trade-offs with each other as a way to maximise fitness among different species (Stearns, 1989; Harvanek et al., 2017; Engl et al., 2020). The trade-off between lifespan and reproduction is a common strategy to maximise evolutionary potential under various selective pressures, such as resource availability constraints. For instance, the more restricted host range of *C. cosyra*, when compared to other *Ceratits* species in Southern Africa (De Villiers et al., 2013), has likely resulted in frequent host availability constraints. It is possible that *C. cosyra*, like other longer-lived species, evolved a longer lifespan to better match their host phenology (Malod et al., 2020). However, the development of a prolonged lifespan comes with constraints on reproductive potential, as governed by the lifespan-reproduction trade-off (Harshman and Zera, 2007). The lower overall fecundity and fertility in the longer-lived *C. cosyra*, in contrast with *C. capitata*, found in this study support the theory of a trade-off between lifespan and reproduction. This indicates that, unlike *C. capitata*, *C. cosyra* prioritises future reproduction and survival efforts over current reproductive potential. Additionally, the survival of reproductively active *C. cosyra* and *C*. capitata in this study contrasts with the much higher survival for virgin females reported by Malod et al. (2020). This further supports the trade-off that occurs between lifespan and reproduction in these species and the substantial cost to reproduction for both *C. cosyra* and *C. capitata* (Carey et al., 1998; Müller et al., 2001; Carey and Molleman, 2010).

Greater numbers of sperm stored directly after mating, followed by subsequent declines in mating propensity and duration indicate that initial sperm transfer by males is enough to partially inhibit remating in both species. Declines in mating propensity alone indicate that mating itself inhibits remating, at least in the short term. The similarity in mating propensity between species further suggests that this inhibition by ejaculate transfer functions to reduce, but not completely inhibit, remating in both species. Short term remating inhibition in *C. capitata* has been previously attributed to the effect of the sperm itself (Nakagawa et al., 1971; Miyatake et al., 1999), with declines in sperm storage resulting in higher occurrences of remating. However, no significant increase in mating propensity was found in either species in this study, despite the decline in sperm storage. Long-term inhibition of mating in *C. capitata* has been associated with accessory gland proteins (AGPs; Miyatake et al., 1999; Mossinson and Yuval, 2003). It is thus likely that initial mating inhibition in this study was due to the presence of stored sperm, while prolonged mating inhibition was due to the presence of inhibitory AGPs. The effect of AGPs on mating inhibition has likewise been found in other tephritids, such as *Anastrepha fraterculus*, *Ceratitis capitata*, *Bactrocera cucurbitae* and *Bactorcera tryoni* (Kuba and Itô, 1993; Jang et al., 1998; Radhakrishnan and Taylor, 2007; Abraham et al., 2012). However, AGPs alone do not inhibit mating in all tephritids, such as in *Anastrepha ludens*, where it has been proposed that the mechanical or even the physiological effect of the full ejaculate that inhibits remating (Abraham et al., 2014). Our findings indicate a combined effect of sperm and AGPs on remating inhibition in *C. cosyra*. Females in the remating study were repeatedly exposed to males, which may have created a habituation effect. This habituation could have led to an underestimate of females’ willingness to remate in this study. Considering this, our results may represent the lower threshold of females’ willingness to remate for these species. Furthermore, the repeated exposure to males for remating may mask the full extent of factors that regulate remating in both the short- and long-term. Future studies could offer only a single male to mated females, at varying intervals, to elucidate how receptivity changes over time and how this is controlled by different regulating factors. The physiological mechanism of remating inhibition in this species is not fully understood, and other factors, such as mate and ejaculate quality, should also be investigated in future studies.

As expected, *C. cosyra* transferred large numbers of sperm during mating. Large sperm transfers are commonly found in species with long copula durations (Thomas et al., 2014). Copula duration in *C. cosyra* in the present study is similar to that recorded by Roets et al. (2018) and is among the longest of all tephritids (Taylor et al., 2000; Aluja et al., 2001; Pérez-Staples and Aluja, 2004; Perez-Staples et al., 2008, 2010). It is thus likely that large sperm transfers occurred due to the long copula duration. Despite this, sperm storage in *C. cosyra* drastically declined by 14 days after mating, with no simultaneous change in fecundity or fertility. One explanation for this unexpected pattern in sperm storage is that large ejaculates, with high numbers of sperm are transferred to the female as a nuptial gift. As suggested to occur in *C. capitata*, this nuptial gift given to *C. cosyra* may be degraded as a source of nutrients to provide adequate energy for future reproductive efforts (Taylor and Yuval, 1999). The transfer of a nuptial gift in this manner may be a form of sexual selection, with females prioritising later reproductive efforts according to the quality and quantity of the transferred ejaculate (Boggs, 1995). Similar postcopulatory selection has also been observed in an arctiid moth, *Utetheisa ornatrix* (Lamunyon and Eisner, 1993). Additionally, the large decline in sperm storage, coupled with low fertility, could be due to females discarding non-viable or damaged sperm. Although females can modulate the metabolism of stored sperm to reduce oxidative damage (Ribou and Reinhardt, 2012), *C. cosyra* females could exhibit sperm dumping to limit the toxicity that results from long-term storage in spermathecae. Furthermore, changes in the number of sperm within the spermathecae may be a result of sperm being transferred to ancillary sperm storage organs, such as the ventral receptacle (Marchini et al., 2001; Fritz and Turner, 2002; Fritz, 2004). It is possible that *C. cosyra* stores a larger proportion of the ejaculate in this incipient organ than *C. capitata*, thus accounting for the large drop in sperm stores seen in *C. cosyra*.

Similar studies have reported higher fertility in *C. capitata* than what was found in the current study (Blay and Yuval, 1999; Twig and Yuval, 2005). It is possible that fertility has thus also been underestimated for *C. cosyra*. Due to oviposition substrates being replaced weekly, lower than expected fertility may be a result of egg desiccation from eggs that were laid outside of the oviposition substrate or that did not fall into the solution within. Despite this, further research is needed on the viability and quality of sperm in *C. cosyra* to determine the cause of lower than expected fecundity and fertility. Additional research on sperm viability in *C. cosyra* could also elucidate whether large declines in sperm storage are a result of the discarding of non-viable sperm by females.

Both species investigated in this study pose a serious threat to South Africa’s fruit industry, with *C. cosyra* causing significant losses to the country’s mango industry (Grové et al., 2006). *Ceratitis capitata* is currently being managed in South Africa through the combined use of the sterile insect technique (SIT; Barnes et al., 2015) and female-targeted bait stations. This research indicates that *C. cosyra* and *C. capitata* have a similar willingness to remate. However, the partial inhibition of remating in *C. cosyra* indicates that the continuous release of sterile males would be needed for the success of SIT programs with this species, due to the importance of mating inhibition to the success of this control method (Miyatake et al., 1999; Kraaijeveld and Chapman, 2004). The willingness of some *C. cosyra* females to remate indicates that existing control through bait stations and development of lures might be a more effective method of control for this pest species. This study also highlights the diversity of life histories at low taxonomic levels and provides more information on the link between life-history traits. Despite new insights gained into the reproductive behaviour of *C. cosyra*, this study also underlines the gaps in knowledge of several aspects of its reproductive biology, namely patterns of sperm storage and sperm viability.

In conclusion, *C. cosyra* had a higher survival and a lower fecundity when compared with the shorter-lived *C. capitata*, but with a similar propensity to remate. This indicates that *C. cosyra* does prioritise future reproductive efforts and survival over current reproduction. The prediction of high initial sperm transfers to female *C. cosyra* was found to be accurate. However, declines in sperm storage and consistent willingness to remate indicate that *C. cosyra* does not store sperm for long periods of time to promote future reproductive success. This study further supports the theory of the lifespan-reproduction trade-off by presenting circumstantial evidence to the cost of reproduction in *C. cosyra*.

## Data Availability Statement

Data for this study are openly available in figshare at http://doi.org/10.25403/UPresearchdata.18669434, reference number 18669434.

## Author Contributions

CW and KM conceived and designed the study. TP conducted the experiments and data collection and wrote the manuscript. TP and CW performed the statistical analyses. CW, KM, and TP read and approved submission of the manuscript.

## Conflict of Interest

The authors declare that the research was conducted in the absence of any commercial or financial relationships that could be construed as a potential conflict of interest.

## Publisher’s Note

All claims expressed in this article are solely those of the authors and do not necessarily represent those of their affiliated organizations, or those of the publisher, the editors and the reviewers. Any product that may be evaluated in this article, or claim that may be made by its manufacturer, is not guaranteed or endorsed by the publisher.
